# Loss of tumorigenic potential upon transdifferentiation from keratinocytic into melanocytic lineage

**DOI:** 10.1038/srep28891

**Published:** 2016-07-08

**Authors:** Sabrina Fehrenbach, Daniel Novak, Mathias Bernhardt, Lionel Larribere, Petra Boukamp, Viktor Umansky, Jochen Utikal

**Affiliations:** 1 Skin Cancer Unit, German Cancer Research Center (DKFZ), 69121 Heidelberg, Germany; 2 Department of Dermatology, Venereology and Allergology, University Medical Center Mannheim, Ruprecht-Karl University of Heidelberg, 68167 Mannheim, Germany; 3 Genetics of Skin Carcinogenesis, German Cancer Research Center (DKFZ), 69121 Heidelberg, Germany; 4 IUF–Leibniz Research Institute for Environmental Medicine, 40021 Düsseldorf, Germany

## Abstract

Lineage-specific transcription factors determine the cell fate during development. Direct conversion of several cell types into other lineages has been achieved by the overexpression of specific transcription factors. Even cancer cells have been demonstrated to be amenable to transdifferentiation. Here, we identified a distinct set of transcription factors, which are sufficient to transform cells of the keratinocytic lineage to melanocyte-like cells. Melanocyte marker expression was induced and melanosome formation was observed in non-tumorigenic keratinocytes (HaCaT) and tumorigenic squamous cell carcinoma (MET-4) cells. Moreover, reduced proliferation, cell metabolism, invasion and migration were measured *in vitro* in transdifferentiated MT-MET-4 cells. A loss of tumorigenic potential of squamous cell carcinoma cells could be due to the upregulation of the melanocyte differentiation associated gene IL-24. Our data show that cells from the keratinocytic lineage can be transdifferented into the melanocytic lineage and provide a proof of principle for a potential new therapeutic strategy.

The direct conversion of one somatic cell type into another mature cell type is called transdifferentiation^1^,^2^,^3^. Cells lose the characteristics of the parental cell type and gain the phenotype of another cell type. Until today, several different cell types have been generated by transdifferentiation. Direct conversion from cells to different cell types arising from the same germ layer^4^,^5^,^6^,^7^ as well as direct conversions across different germ layers^8^,^9^,^10^,^11^ were achieved. First attempts to generate melanocytes by transdifferentiation have been made by ectopically overexpressing MITF in murine fibroblasts. These cells adopted melanocytic characteristics; however, fully functional melanocytes could not be generated^12^. Recently, the three factor combination MITF, SOX10 and PAX3 was identified to be sufficient to convert murine and human fibroblasts into mature melanocytes^13^. In 2013, Thomas Graf and colleagues succeeded in transdifferentiating cancer cells^14^. Ectopic overexpression of the transcription factor C/EBPα in tumorigenic B cell lymphoma and leukemia cells resulted in their conversion into functional macrophages. The expression of C/EBPα led to the downregulation of B cell markers, expression of macrophage markers and gain of macrophage characteristics including increased adherence, granularity and cell size. In addition, the cells acquired high phagocytic activity. Remarkably, the converted cells also showed significantly impaired tumorigenic potential after injection into immunodeficient mice^14^. Interestingly, in contrast to reprogramming, methylation changes seem to play a minor role in transdifferentiation as identified by Rodríguez-Ubreva *et al*.^15^.

Here, we demonstrate that non-tumorigenic and cancer cells from the keratinocytic lineage (HaCaT and MET-4 cells) can be transdifferentiated towards the melanocytic lineage using distinct transcription factor combinations. In addition to adopting melanocyte characteristics, transdifferentiation of squamous cell carcinoma (SCC) cells resulted in a loss of their tumorigenic potential.

## Results

### Identification of melanocyte transdifferentiation factors

**Figure 1:**
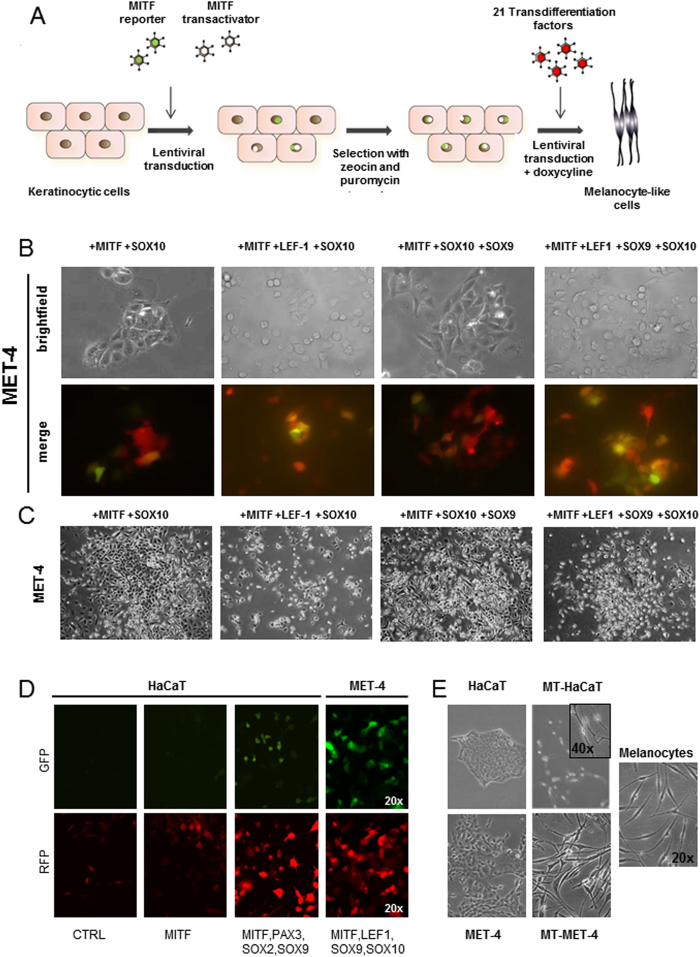
Transdifferentiation of HaCaT and MET-4 cells into melanocyte-like cells. (**A**) Scheme of workflow. Cells were first selected for the M2 transactivator and MITF reporter and afterwards transduced with transcription factor cocktail and induced with doxycycline (**B**) Transgene expression was confirmed with different transcription factor combinations. Stronger RFP signal was observed depending on the amount of different factors used. Various transcription factor cocktails could induce MITF expression in MET-4 cells. The strongest expression was noted for the factor combination MITF, LEF-1, SOX9 and SOX10. (**C**) Different transcription factor combinations could induce morphological changes in MET-4 cells. The cells changed their morphology from cobblestone-like appearance into more spindle-shaped cells. Cell-cell adhesion was lost and colony formation was reduced. Single cells with loose contacts to the neighbouring cells were present. (**D**) Transgene expression (RFP) and MITF reporter activation (GFP) in HaCaT and MET-4 cells for different factor combinations (**E**) Morphology of HaCaT cells, MET-4 cells and melanocytes as well as morphological changes observed after induction of the transdifferentiation factor overexpression in melanocyte-transdifferentiated HaCaT (MT-HaCaT) and melanocyte-transdifferentiated MET-4 (MT-MET-4) cells.

To induce transdifferentiation, 21 candidate transcription factors (MITF-M, SOX2, SOX5, SOX9, SOX10, LEF1, Beta-catenin, PAX3, SNAI2, cMET, TFAP2A, NFIX, IRF4, ETS1, FOSB, HAND1, HES1, HOXB7, IFI16, KLF9, PITX1) known to be involved in neural crest development and melanocyte differentiation were cloned into doxycycline-inducible lentiviral vectors (Figure S1A). First, cells of the keratinocytic lineage were transduced with a reverse tetracycline-controlled transactivator (M2) and a lentiviral melanocyte-specific reporter construct, harboring GFP under the control of the MITF-M promoter (Figure S1A), and selected for these two vectors with zeocin and puromycin, respectively. After transduction with the lentiviral particles carrying the transdifferentiation factors, expression was induced with doxycycline (Fig. 1A). Transgene expression was indicated by RFP co-expression from the same construct. Transdifferentiation towards the melanocytic lineage was monitored by GFP expression from the reporter construct. The functionality of the reporter construct was tested using melanocytes, HaCaT cells and MET-4 cells. In melanocytes a strong GFP signal could be observed while no reporter activity was detected in the other two cell lines of the keratinocytic lineage (Figure S1B). After two to three days of treatment with doxycycline, transgene expression could be noted. Within a week of treatment, MITF reporter expression was detected. MITF alone did not activate reporter activity. Several factor combinations were able to activate MITF reporter activity in MET-4 cells. Strongest GFP fluorescence was observed with the factor combination MITF, LEF1, SOX9 and SOX10. The most effective factor combination for HaCaT cells was MITF, PAX3, SOX2 and SOX9. (Fig. 1B,D; Figure S2A).

Ectopic expression of MITF was reported to be sufficient to induce melanocyte characteristics in fibroblasts^12^. However, in MET-4 cells, expression of MITF alone did not induce morphological changes in these cells (Figure S2A). Non-induced control cells did not show any transgene expression. To support transdifferentiation, cells were cultured in MCDB medium, which favors the growth of melanocytes whereas non-treated MET-4 cells did not survive under these culture conditions (Figure S2A). Morphological changes could be observed within 10 days after induction. MET-4 cells have a cobblestone-like appearance which changed into spindle-shaped morphology which is typical for melanocytes. Additionally the cells lost the close contact to the neighboring cells (Fig. 1C,E). The cells retained the melanocyte morphology also after doxycycline withdrawal without detectable transgene expression. The cells could be cultured over 20 passages without doxycycline and still kept the spindle-shaped morphology indicating stable transdifferentiation (Figure S2B). Cells with melanocyte morphology (MT-MET-4) were enriched by serial trypsinization since these cells detached much faster than the parental MET-4 cells.

### HaCaT and MET-4 cells adopt melanocytic characteristics

**Figure 2:**
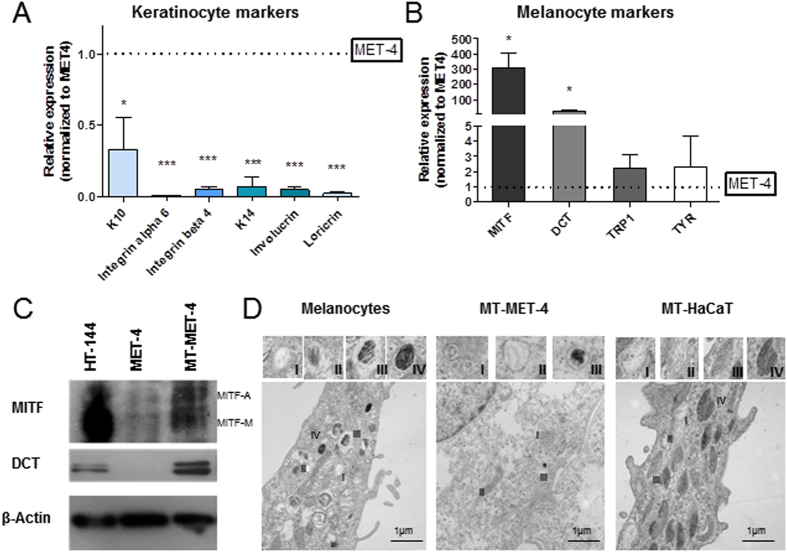
Melanocyte characteristics in MT-MET-4 cells and MT-HaCaT cells. (**A**) Expression of keratinocyte markers and (**B**) melanocyte markers in transdifferentiated MT-MET-4 cells compared to MET-4 cells. Several keratinocyte markers were downregulated in the MT-MET-4 cells relative to the MET-4 cells. Error bars show SD of three independent experiments. P values were calculated by two-tailed, unpaired sample t-test (*p < 0.05, **p < 0.01, ***p < 0.005). (**C**) Western Blot analysis of the expression of the melanocyte markers MITF and DCT. (**D**) Electron microscope pictures showing stage I to IV of melanosomes in human melanocytes. Melanosomes in stage I-III were present in MT-MET-4 cells. Transdifferentiated HaCaT cells contained high amount of melanosomes from all four stages.

**Figure 3:**
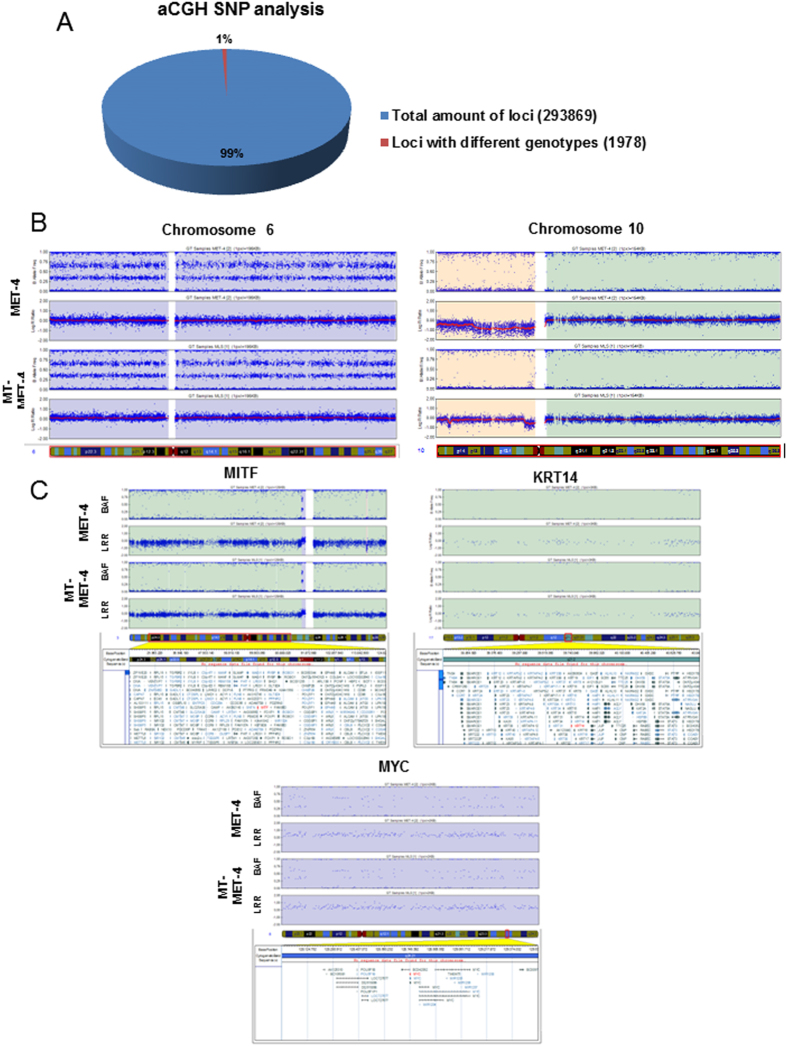
Analysis of chromosomal abberations. (**A**) aCGH SNP analysis. The genotypes of 293869 loci in MET-4 and MT-MET-4 were compared. The same genotype was identified for 99% of the loci analysed. (**B**) Most of the genomic regions in MET-4 and MT-MET-4 were present in the same copy number. Chromosome 6 and 10 are shown representatively. (**C**) The copy number variation has been compared for several genes of interest. No variation was present in melanocyte-specific genes, keratinocyte-specific genes or tumor suppressors and oncogenes. Shown here representatively are MITF, KRT14, and MYC.

**Figure 4:**
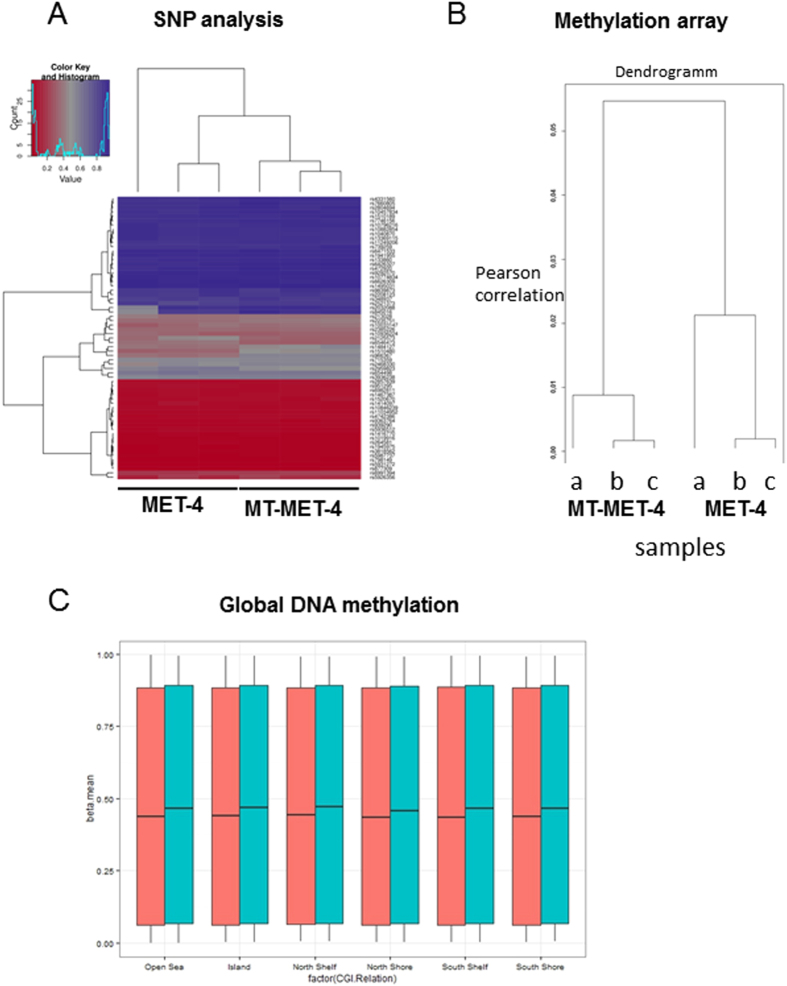
Methylation array analysis. (**A**) Methylation array SNP analysis. MET-4 and MT-MET-4 cells show extremely similar SNP profiles confirming that MT-MET-4 are indeed derived from MET-4 cells. (**B**) Dendrogram of methylation array analysis. All three MET-4 samples cluster together and all three MT-MET-4 samples cluster together. (**C**) Global DNA methylation. A higher overall methylation was found in MT-MET-4 cells compared to MET-4 throughout the genome.

In transdifferentiated cells, the keratinocyte markers Keratin10 (K10), Keratin14 (K14), Integrin alpha 6, Integrin beta 4, Involucrin and Loricrin were downregulated while the melanocyte markers MITF, DCT, TRP-1 and tyrosinase were upregulated (Fig. 2A,B). Western Blot analysis confirmed the expression of MITF and DCT in MT-MET-4 cells at the protein level (Fig. 2C). Via electron microscopy, spherical pheomelanosom-like structures were visible in transdifferentiated MT-MET-4 cells and melanosome-like structures were detected in transdifferentiated HaCaT cells but not in the parental cells (Fig. 2D). To demonstrate that genomic alterations are not responsible for the observed phenotype an array CGH screen was performed. Analysis of these data showed that over 99% of the analyzed loci had the same genotype (Fig. 3A). An additional global SNP analysis demonstrated that MET-4 cells and MT-MET-4 cells cannot be distinguished at the genomic level (Fig. 4A). In general, high amount of chromosomal abnormalities was noted for both cell types. Copy number variation analysis (CNV) demonstrated that most of the sequences were present in the same copy number (Fig. 3B,C). In addition, the loci of melanocyte- or keratinocyte-related genes as well as of oncogenes including c-Myc and tumor suppressor genes such as p53 did not show any copy number variation (Fig. 3B,C). We, therefore, hypothesized that epigenetic changes were responsible for the phenotype. Performing methylation profiles, the parental and the transdifferentiated cells could be clearly separated from each other. The dendrogram received after quantile normalization of all samples (3 samples for MET4 cells, 3 samples for MT-MET-4 cells) depicted that the distance between them was very low with a distance expressed in Pearson correlation of 0.06 (Fig. 4B). The global methylation analysis revealed a general increase in methylation in MT-MET-4 cells compared to the parental cells at every location which was analyzed (Fig. 4C).

### Transdifferentiation leads to loss of tumor forming potential in MT-MET-4 cells

**Figure 5:**
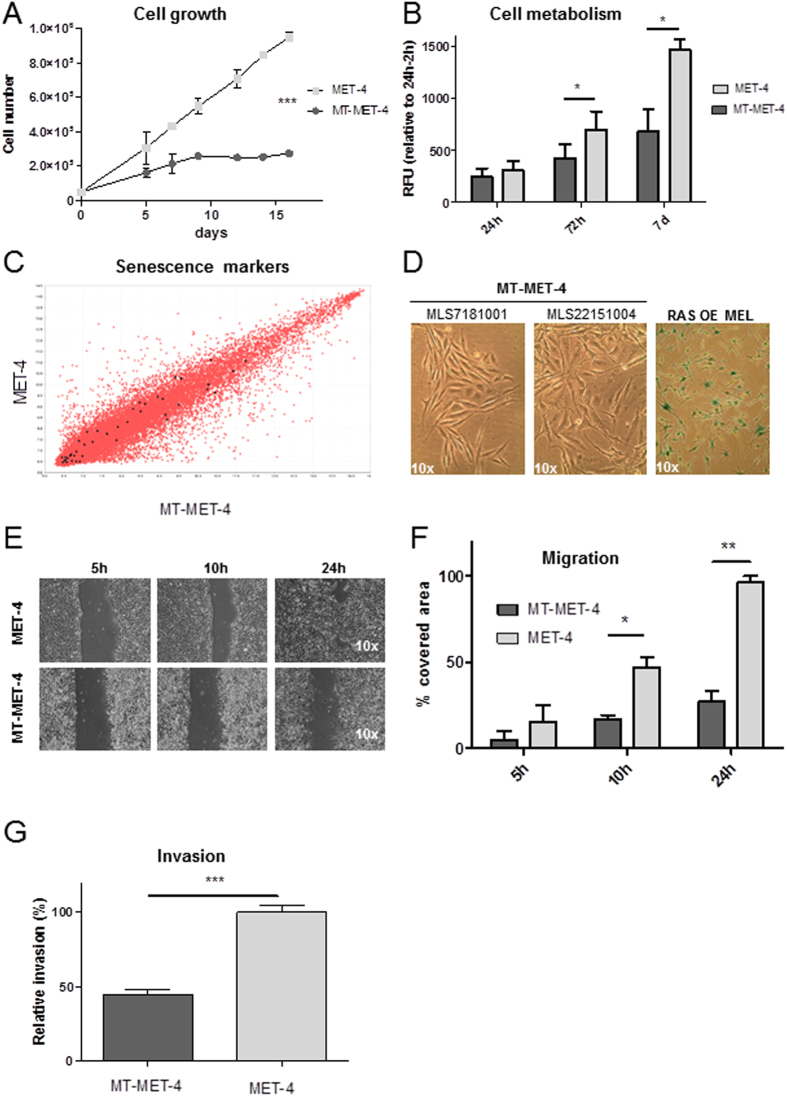
Analysis of transdifferentiated MT-MET-4 cells. (**A**) Cell growth curve. Error bars depict the SD of three independent experiments. P values were calculated by two-way ANOVA (***p < 0.005). **(B**) Cell metabolism analysis. The metabolic activity of MET-4 and MT-MET-4 cells was measured 24 h, 72 h and 7d after seeding. (**C**) Comparison of senescence marker expression in MET-4 cells and MT-MET-4 cells. (**D**) β-galactosidase staining of two different batches of MT-MET-4 cells (MLS7181001 and MLS22151004) and RAS-overexpressing normal human melanocytes (RAS OE MEL). In comparison to the melanocytes, the MT-MET-4 cells are negative for the senescence marker β-galactosidase. (**E**) Migration assay. The migration capacity of MET-4 cells and MT-MET-4 cells was observed over 24 h. (**F**) Quantification of the migration potential. For analysis the software t-scratch was used which calculated the % of free area. (**G**) Invasion assay. MT-MET-4 cells are less invasive compared to the parental MET-4 cells.

**Figure 6:**
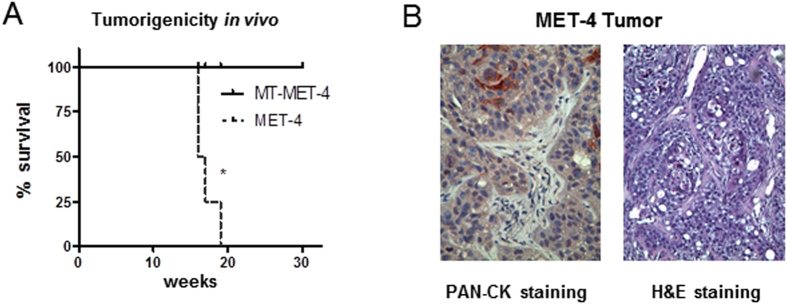
Tumorigenic potential of MET-4 and MT-MET-4 cells. (**A**) Overview of tumorigenic potential *in vivo*. Subcutaneous injection of MET-4 cells in the flanks of NSG mice led to tumor formation in all four mice (n = 4). No tumor growth was observed for MT-MET-4 cells. (**B**) PAN-CK and H&E staining of the MET-4 tumor.

To test if the tumor forming capacity of the cells was reduced after transdifferentiation several characteristics related to malignant phenotype such as proliferation and cell motility were investigated *in vivo*. As measurement for proliferation MET-4 cells and MT-MET-4 cells were counted over a period of 16 days. While a linear growth curve was observed for MET-4 cells with almost 1 × 10^6^ cells at the end of observation, only slight increase in cell number was seen for MT-MET-4 cells finally counting 2 × 10^5^ cells. Thus, transdifferentiated cells divided less frequently than the parental cells. Further, MT-MET-4 cells showed a significantly reduced cell metabolism in comparison to MET-4 cells measured 72 h and 7d after seeding (Fig. 5A,B). To exclude that induction of senescence might be responsible for the reduced growth potential and the decreased metabolic activity senescence marker expression was analyzed. No significant changes in expression were observed comparing MET-4 cells with MT-MET-4 cells (Fig. 5C, Figure S3A,B). Beyond that, staining against the senescence marker β-galactosidase was negative in MT-MET-4 cells in contrast to RAS-overexpressing melanocytes indicating the absence of this senescence marker in the transdifferentiated cells (Fig. 5D). Additionally, a migration and an invasion assay were performed. The migratory potential was strongly reduced. After 24 h, MT-MET-4 cells covered about 20% of the free area while the parental MET-4 cells covered more than 90% of the free area (Fig. 5E**,F**). A transwell assay with 0,1x BME coating demonstrated that the transdifferentiated cells were approximately 50% less invasive in comparison to the parental MET-4 cells as well (Fig. 5G). Finally, we assayed the tumorigenicity by injecting the MET-4 cells (1.5 × 10^6^) s.c. into the flanks of NSG mice (n = 4). While MET-4 cells induced tumors in all four mice injected within 16–18 weeks, no tumor developed over a period of 30 weeks when the transdifferentiated MT-MET-4 cells were injected (Fig. 6A,B).

### Upregulation of anticancer gene IL-24 in transdifferentiated melanocytic cells

**Figure 7:**
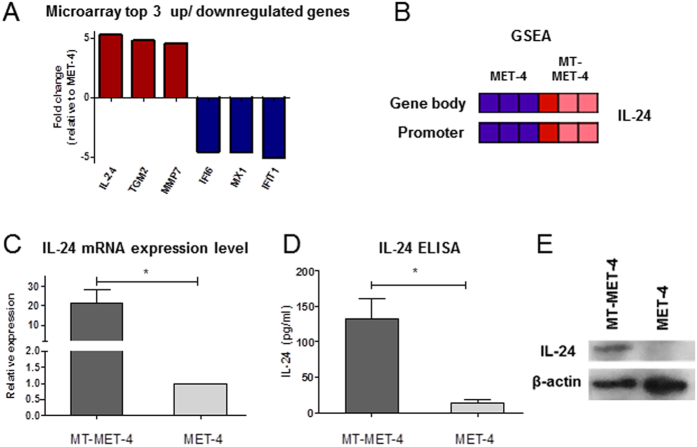
Validation of IL-24 expression. (**A**) Microarray top 3 up/downregulated genes (**B**) Gene set enrichment analysis of differentially methylated genes and whole genome expression array. IL-24 was a top hit candidate after this analysis (**C**) IL-24 expression on mRNA level. Increased expression of IL-24 in MT-MET-4 cells was confirmed via Q-PCR. Error bars depict the SD of three independent experiments. P values were calculated by two-tailed, unpaired sample t-test (*p < 0.05). (**D,E**) IL-24 expression on protein level. Secretion of the IL-24 protein and intracellular IL-24 protein measured in supernatant and cell lysate of MET-4 and MT-MET-4, respectively. Error bars depict the SD of three independent experiments. P values were calculated by two-tailed, paired sample t-test. (*p < 0.05, **p < 0.01, ***p < 0.005).

To determine the mechanism which could explain the impaired tumorigenic potential in the transdifferentiated MT-MET-4 cells, RNA expression array analysis was performed. The Microarray data suggested differential regulation of several genes between MET-4 and MT-MET-4 cells. The top three up- and downregulated genes (up: IL-24, TGM2, MMP7, down: IFI6, MX1, IFIT1) are depicted in Fig. 7A. Gene set enrichment analysis (GSEA) of the expression array data combined with the methylation array data identified IL-24 as a top hit to be highly upregulated and its promoter and gene body to be less methylated in the MT-MET-4 cells (Fig. 7B). Higher expression of IL-24 could be confirmed with Q-PCR (Fig. 7C) and higher concentration of IL-24 protein was measured in the supernatant of MT-MET-4 cell cultures (Fig. 7D) as well as in the cell lysate using Western Blot analysis (Fig. 7E). Analysis of IL-24 mRNA expression in 4 melanoma cell lines (A375, SK-MEL23, SK-MEL30, SK-MEL28) and primary melanocytes revealed a reduction in expression.

**Figure 8:**
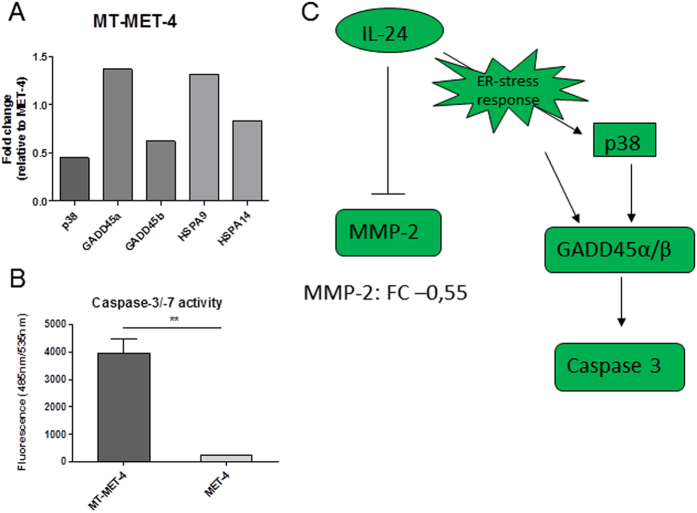
Functional mechanism of IL-24 in MT-MET-4 cells. (**A**) Gene expression of MAPK p38, GADD family members 45 α/β, and HSPA9 and HSPA14 (**B**) Caspase-3/7 assay for MET-4 and MT-MET-4 cells (**C**) Schematic representation of the signalling pathway regulated by IL-24 in MT-MET-4 cells.

Detailed analysis of the gene expression data comparing MET-4 with MT-MET-4 revealed an upregulation of MAPK p38 and two GADD family members (GADD 45α/β) in the transdifferentiated MT-MET-4 cells. Additionally, two heat shock proteins (HSPA9, HSPA14) known to be involved in ER-stress response were found to be upregulated in MT-MET-4 cells (Fig. 8A). A caspase-3/7 assay demonstrated increased caspase activity in the MT-MET-4 cells compared to MET-4 cells (Fig. 8B). Further, the gene expression array indicated a downregulation of MMP-2 in MT-MET-4 cells relative to MET-4 cells. In summary, we could show that IL-24 leads to ER-stress in the cells which activates caspases via MAPK p38 and members of the GADD family. This pathway is known to be upregulated in melanoma cells resulting in cell cycle arrest and growth inhibition. Via downregulation of MMP-2 the migration and invasion potential might be significantly reduced in MT-MET-4 cells (Fig. 8C).

12-O-tetradecanoylphorbol 13-acetate (TPA), which is a component of our melanocyte-specific MCDB medium, was described to induce IL-24 expression^16^. In our study we observed a slight increase in the IL-24 expression when MT-MET-4 cells were cultured with TPA compared to the cultured untreated cells. However, the IL-24 expression of MT-MET-4 cells cultured without TPA was still significantly higher than of the MET-4 cells (Figure S4) indicating that the elevated level of IL-24 is in part due to the transdifferentiation.

## Discussion

Transdifferentiation, the direct conversion of one somatic cell type into another, is a promising approach to uncover basic principles of differentiation and development and could prove very useful for tissue replacement therapy and drug screening. Yang *et al*.^13^ have recently succeeded in generating melanocytic cells from murine and human fibroblasts by ectopically overexpressing the three factors MITF, SOX10, and PAX3. Moreover, they demonstrated that also other combinations of transcription factors could induce transdifferentiation towards the melanocytic lineage albeit with less efficiency. In our study, we could show that cells from the keratinocyte lineage (ectodermal) are also amenable to conversion into melanocyte-like cells by ectopically overexpressing a defined factor combination different from those used in the above mentioned work^13^. The fact that different transcription factor combinations efficiently generate melanocyte-like cells may be due to overlapping functions of the used factors or different somatic cell types used as the starting material. MITF seemed to be essential for the conversion into melanocyte-like cells. In addition, factors such as PAX3, SOX2, SOX9, SOX10 and LEF-1 were necessary for the transdifferentiation depending on the cell type of origin. For conversion of fibroblasts MITF combined with SOX10 and PAX3 showed the strongest effect in inducing melanocytic characteristics. In HaCaT cells MITF together with PAX3, SOX2 and SOX9 stimulated melanosome synthesis while in MET-4 cells the ectopic expression of MITF, SOX9, SOX10 and LEF1 activated the melanocytic phenotype. The transdifferentiated cells obtained in our study adapted a spindle-shaped morphology similar to melanocytes, downregulated keratinocytic markers, expressed melanocytic markers and activated melanogenesis. Beyond that, the conversion of squamous cell carcinoma cells to melanocyte-like cells markedly impaired their tumorigenic potential. Our findings support the hypothesis that the tumorigenicity of cancer cells is dependent on their differentiation status and linked to a specific lineage^14^. It was shown that B cell leukemia and lymphoma cells can be converted into macrophages, and that after injection of these macrophages into immunodeficient mice reduced tumor formation was observed while the parental cell lines were 100% lethal under the same conditions^14^. In our hands, the MET-4 cells, which were derived from a cutaneous SCC metastasis and which are highly tumorigenic^17^ lost their tumorigenic potential upon transdifferentiation. Thus, even genetically-driven tumorigenicity is “reversible” when the cells face a different endogenous regulatory network. We observed an upregulation of IL-24 in the transdifferentiated MT-MET4 cells. This cytokine is normally expressed in melanocytes, but is often lost upon malignant transformation into melanoma. Interestingly, the IL-24 level is again upregulated upon forced differentiation of melanoma cells^18^. Keratinocytes compared to melanocytes show only a weak expression^19^. IL-24 is known to inhibit tumor growth, invasion, metastasis and angiogenesis in different tumors including melanoma while no toxic effect was observed in the normal somatic counterpart^20^,^21^. We hypothesize that the conversion of tumor cells of keratinocytic origin to melanocyte-like cells overrides their tumorigenic phenotype by two different mechanisms. On one hand, the loss of keratinocyte-specific features goes along with a downregulation of keratinocyte-specific signaling pathways that ran out of control in MET-4 cells. On the other hand, the increasing melanocyte character activates melanocyte-specific signaling routes. One of these signaling routes employs IL-24 as an autocrine messenger, which contributes to reducing the tumorigenicity and proliferative potential of the former squamous cell carcinoma cells. We suggest that these data provide a proof of principle for a potential new therapeutic strategy in the future.

## Material and Methods

### Cell lines

The immortalized human keratinocyte cell line HaCaT^22^ and the human SCC cell line MET-4^17^,^23^ were cultured at 37 °C with 5% CO_2_ in Dulbecco’s Modified Eagle Medium (DMEM) (Gibco^®^ Life Technologies) with 10% FCS (Biochrom) and 1% Pen/Strep (Sigma-Aldrich). HT-144 melanoma cells were cultured in DMEM with 10% FCS, 0.1 mM β-mercaptoethanol (Gibco^®^ Life Technologies), and Pen/Strep. Human melanocytes were isolated from foreskins and cultivated in medium 254 (Gibco^®^ Life Technologies) supplemented with 1% 100x human melanocyte growth supplement (HMGS) (Gibco^®^ Life Technologies) with a final concentration of 0.2% bovine pituitary extract, 0.5% FBS, 1 μg/ml recombinant human insulin-like growth factor-I, 5 μg/ml bovine transferrin, 3 ng/ml basic fibroblast growth factor (bFGF), 0.18 μg/ml hydrocortisone, 3 μg/ml heparin, and 10 ng/ml phorbol 12-myristate 13-acetate (PMA). Experiments were carried out in accordance with the approved guidelines. All experimental protocols were approved by the ethics committee II of the University of Heidelberg (2009-350N-MA). Informed consent was obtained from all subjects.

Transdifferentiated cells were cultured in MCDB medium (Sigma-Aldrich) supplemented with 2% FCS, 0,02 mg/ml bovine Pituitary Extract (bPE) (Gibco^®^ Life Technologies), 0.001 μg/ml basic fibroblast growth factor (bFGF) (Promokine), 0.005 mg/ml insulin (Sigma-Aldrich), 0.0005 mg/ml hydrocortisone (Sigma-Aldrich), 0.01 mM forskolin (Tocris) and in the presence or absence of 0.016 μM 12-O-tetradecanoylphorbol 13-acetate (TPA) (Sigma-Aldrich).

### Cloning of the constructs and viral vector production

The human MITF promoter was amplified from the plasmid pMI, kindly provided by Dr. Ballotti, Nice^24^, with the following primers:

MITF prom forward → CGCATCGATAGGCCGTTAGAAACATGATC

MITF prom reverse → CGCTCTAGACAATCCAGTGAGAGACGGTAG

The promoter was then cloned into pLenti CMV GFP Puro (pLenti CMV GFP Puro (658-5) which was a gift from Eric Campeau; Addgene plasmid # 17448;^25^). The CMV promoter was cut from pLenti CMV GFP Puro with Bsu15I and XbaI and replaced with the MITF promoter.

Candidate transcription factors (Suppl. Table 1) were amplified by PCR or cut from their original vectors and introduced into the MCS-IRES-RFP vector (MIR). MIR was generated from tetO-human cMyc-IRES-GFP (kindly provided by Dr. von Kalle, (NCT, Heidelberg)) by replacing cMyc-IRES-GFP with a cassette comprising a multiple cloning site, IRES and RFP. pcDNA3.1/myc-His (−) B –hMITF-M was a gift from Dr. Esumi, Baltimore. pCMV/huSOX10 was kindly provided by M. Wegner^26^. pEGFP-C1 human AP-2α was made available by Dr. Godbout^27^. EF.hHES1.Ubc.GFP was a gift from Linzhao Cheng (Addgene plasmid # 17624). Expression plasmids containing the ORFs from SOX9, ETS1, FOSB, HAND1, HOXB7, IFI16, KLF9, NFIX and PITX1 were purchased from the DNASU plasmid repository.

Lentiviral particles were prepared by transfecting HEK293T with lentiviral vectors and packaging constructs pCMV-dR8.91 and pCMV-VSV-G with XtremeGENE 9 (Roche). Virus-containing supernatants were collected after 36, 48 and 72 hr.

### RNA isolation, cDNA synthesis and quantitative RT-PCR analysis

RNA isolation was conducted using the RNeasy Kit supplied by Qiagen following the manufacturer’s protocol. NanoDrop ND-1000 Spectrophotometer was used for RNA concentration and quality measurement. cDNA was synthesized using the RevertAid First Strand cDNA Synthesis Kit (Thermo Scientific) according to the manufacturer’s protocol. 18S was used as reference gene for RT-PCR analysis. Gene quantification was calculated using the Pfaffl method^28^ calculating the delta-delta Ct. Primers used, are shown in Suppl. Table 2.

### Protein extraction and Western Blot

Cells were harvested in PBS and lysed in RIPA lysis buffer (50 mM Tris-HCl pH7.5, 150 mM NaCl, 1% Triton-X-100, Roche Complete Mini Protease Inhibitor Cocktail (EDTA)). The protein concentration was measured using the Pierce BCA Protein Assay kit. The following primary antibody dilutions were used in 5% non-fat dried milk: MITF (abcam ab80651, mouse mAb 1:1000), DCT (protein tech, rabbit pAb 1:1000), IL-24 (abcam ab56811, mouse mAb 2 μg/ml), beta-Actin (Cell Signaling 13E5, rabbit mAb, 1:1000). The secondary antibodies (goat anti-rabbit or goat anti-mouse horseradish peroxidase-linked antibody, Cell Signaling Technology) were diluted 1:10.000 in 5% non-fat dried milk in TBST and incubated at room temperature for 1 h.

### Electron microscopy

Cells were seeded on Aclar^®^ slides. The electron microscopy core facility of the DKFZ performed the chemical fixation, dehydration, embedding and imaging of the samples.

### Cell growth analysis and cell metabolism

Cells were counted over a period of 16 days at 48 h and 72 h intervals. The cell viability was assessed using the Thermo Scientific alamarBlue cell viability assay reagent. After 24 h, 48 h and 72 h, alamarBlue reagent was added in a 1:10 dilution and the fluorescence was measured with a SpectraMax M5 microplate reader at 590 nm 2 h and 4 h after addition of the solution. Cell viability was calculated from the resulting change in fluorescence intensity normalized to the 24 h time point measured 2 h after alamarBlue administration.

### Migration assay and invasion assay

To study the migration potential of the cells, ibidi culture inserts were used. After starvation overnight, the proliferation inhibitor mitomycin C (Carl Roth) was added, the inserts were removed and migration was observed every 5 h over 24 h for MET-4 cells and over 96 h for MT-MET-4 cells. Quantitative data analysis was performed with the software TScratch. The invasiveness of the cells was analyzed using the Cultrex 24-well BME Cell Invasion Assay (Trevigen) according to the manufacturer’s protocol with 0.1x BME (basal membrane extract). Invading cells were lysed with a dissociation buffer containing Calcein, a fluorescent dye used for quantification. The TECAN infinite F200 pro microplate reader was used to measure fluorescence at 485 nm excitation and 520 nm emission.

### IL-24 ELISA

The human IL-24 ELISA kit from Cusabio was used for measurement of secreted IL-24 according to the manufacturer’s protocol. Optical density was measured at 450 nm with a correction at 570 nm. The software Curve Expert 1.4 was used for analysis.

### β-galactosidase staining

β-galactosidase staining was performed with the Senescence β-Galactosidase Staining Kit from Cell Signalling Technology according to the manufacturer’s protocol.

### Caspase-3/7 assay

Caspase activity in MET-4 and MT-MET-4 cells was measured with the Apo-ONE^®^ Homogeneous Caspase-3/7 Assay from Promega according to the manufacturer’s protocol.

### *In vivo* tumorigenicity assay

To investigate the tumorigenicity *in vivo* 1.5 × 10^6^ cells were resuspended in 50% Matrigel (Corning) and injected subcutaneously into the flanks of NSG mice. The overall observation time for tumor formation was 30 weeks. Mice were sacrificed as soon as the tumor reached a size of 1 cm in diameter. Animal experiments were carried out in accordance with the approved guidelines. All experimental protocols were approved by institutional and licensing committees (Regierungspräsidium Baden-Württemberg (G-105/12)).

### Gene expression profiling by Microarray analysis

Biological RNA triplicates of MET-4 cells and MT-MET-4 cells were sent to microarray analysis using Illumina HumanHT-12v4 Expression BeadChip according to the manufacturer’s instructions, at the Genomics and Proteomics Core Facility at the German Cancer Research Center (DKFZ). Quality control, reverse transcription with labeling, chip hybridization and calculation of mean averages was conducted in the core facility for each probe. Chipster was used for quantile normalization of the raw microarray data. Removal of probes with a fold change <2 and samples missing annotation was performed before analysis.

### DNA isolation and Methylation array analysis

Genomic DNA was isolated from MET-4 and MT-MET-4 cells using the QIAGEN DNeasy Blood & Tissue Kit according to the manufacturer’s instructions. Genome-wide methylation analysis using Illumina Infinium HumanMethylation450 BeadChips according to the manufacturer’s instructions was performed at the German Cancer Research Center (DKFZ) Genomics and Proteomics Core Facility. The software RnBeads was used for analysis and a gene set enrichment analysis was conducted using Gene set enrichment analysis (GSEA) software.

### Array Comparative Genome Hybridization (aCGH)

An aCGH was performed for comparison of copy number variations. Genomic DNA was isolated as already described and sent to the Genomics and Proteomics Core Facility of the DKFZ. Samples were analyzed with the HumanCytoSNP-12 (SNP-Array with genome wide coverage) (Illumina) according to the manufacturer’s instructions. Data were analysed using Genome Studios.

### Statistical analysis

The software GraphPad Prism version 5.00 was used for statistical analysis. Statistical significance was determined using two-tailed paired and unpaired t-test, two-way ANOVA, or Fisher’s Exact test. Statistical analysis of q-PCR data was carried out in Excel. Microarray data were analyzed after normalization using an empirical Bayes two groups test using the Benjamini-Hochberg (BH) p value adjustment with a threshold of p < 0.05. p < 0.05 was considered statistically significant.

## Additional Information

**How to cite this article**: Fehrenbach, S. *et al*. Loss of tumorigenic potential upon transdifferentiation from keratinocytic into melanocytic lineage. *Sci. Rep.* **6**, 28891; doi: 10.1038/srep28891 (2016).

## Supplementary Material

Supplementary Information

## References

[b1] Slack J. M. & Tosh D. Transdifferentiation and metaplasia-switching cell types. Curr. Opin. Genet. Dev. 11, 581–6 (2001). 11532402 10.1016/s0959-437x(00)00236-7

[b2] Tosh D. & Slack J. M. How cells change their phenotype. Nat. Rev. Mol. Cell Bio. 3, 187–94 (2002). 11994739 10.1038/nrm761

[b3] Boukamp P. Transdifferentiation induced by gene transfer. Semin. Cell Bio. 6, 157–63 (1995). 7548855 10.1006/scel.1995.0022

[b4] Song K. . Heart repair by reprogramming non-myocytes with cardiac transcription factors. Nature 485, 599–604 (2012). 22660318 10.1038/nature11139 PMC3367390

[b5] Xie H., Ye M., Feng R. & Graf T. Stepwise reprogramming of B cells into macrophages. Cell 117, 663–76 (2004). 15163413 10.1016/s0092-8674(04)00419-2

[b6] Zhou Q., Brown J., Kanarek A., Rajagopal J. & Melton D. A. *In vivo* reprogramming of adult pancreatic exocrine cells to beta-cells. Nature 455, 627–32 (2008). 18754011 10.1038/nature07314 PMC9011918

[b7] Laiosa C. V., Stadtfeld M., Xie H., de Andres-Aguayo L. & Graf T. Reprogramming of committed T cell progenitors to macrophages and dendritic cells by C/EBP alpha and PU.1 transcription factors. Immunity 25, 731–44 (2006). 17088084 10.1016/j.immuni.2006.09.011

[b8] Novak D., Weina K. & Utikal J. From skin to other cell types of the body. J. Dtsch. Dermatol. Ges. 12, 789–92 (2014). 25087921 10.1111/ddg.12403

[b9] Boukamp P., Chen J., Gonzales F., Jones P. A. & Fusenig N. E. Progressive stages of “transdifferentiation” from epidermal to mesenchymal phenotype induced by MyoD1 transfection, 5-aza-2′-deoxycytidine treatment, and selection for reduced cell attachment in the human keratinocyte line HaCaT. J. Cell Biol. 116, 1257–71 (1992). 1371288 10.1083/jcb.116.5.1257 PMC2289354

[b10] Vierbuchen T. . Direct conversion of fibroblasts to functional neurons by defined factors. Nature 463, 1035–41 (2010). 20107439 10.1038/nature08797 PMC2829121

[b11] Fu L., Zhu X., Yi F., Liu G. H. & Izpisua Belmonte J. C. Regenerative medicine: transdifferentiation *in vivo*. Cell. Res. 24, 141–2 (2014). 24343579 10.1038/cr.2013.165 PMC3915906

[b12] Tachibana M. . Ectopic expression of MITF, a gene for Waardenburg syndrome type 2, converts fibroblasts to cells with melanocyte characteristics. Nat. Genet. 14, 50–4 (1996). 8782819 10.1038/ng0996-50

[b13] Yang R. . Direct conversion of mouse and human fibroblasts to functional melanocytes by defined factors. Nat. Commun. 5, 5807 (2014). 25510211 10.1038/ncomms6807 PMC4335710

[b14] Rapino F. . C/EBPα induces highly efficient macrophage transdifferentiation of B lymphoma and leukemia cell lines and impairs their tumorigenicity. Cell. Rep. 3, 1153–63 (2013). 23545498 10.1016/j.celrep.2013.03.003

[b15] Rodríguez-Ubreva J. . Pre-B cell to macrophage transdifferentiation without significant promoter DNA methylation changes. Nucleic Acids Res. 40, 1954–68 (2012). 22086955 10.1093/nar/gkr1015 PMC3299990

[b16] Sauane M. . Autocrine regulation of mda-7/IL-24 mediates cancer-specific apoptosis. Proc. Natl. Acad. Sci. USA 105, 9763–8 (2008). 18599461 10.1073/pnas.0804089105 PMC2474541

[b17] Proby C. M. . Spontaneous keratinocyte cell lines representing early and advanced stages of malignant transformation of the epidermis. Exp. Dermatol. 9, 104–17 (2000). 10772384 10.1034/j.1600-0625.2000.009002104.x

[b18] Jiang H., Lin J. J., Su Z. Z., Goldstein N. I. & Fisher P. B. Subtraction hybridization identifies a novel melanoma differentiation associated gene, mda-7, modulated during human melanoma differentiation, growth and progression. Oncogene 11, 2477–86 (1995). 8545104

[b19] Reemann P. . Expression of class II cytokine genes in children’s skin. Acta Derm. Venereol. 94, 386–92 (2014). 24284923 10.2340/00015555-1717

[b20] Menezes M. E. . MDA-7/IL-24: multifunctional cancer killing cytokine. Adv. Exp. Med. Biol. 818, 127–53 (2014). 25001534 10.1007/978-1-4471-6458-6_6 PMC4633013

[b21] Panneerselvam J., Munshi A. & Ramesh R. Molecular targets and signaling pathways regulated by interleukin (IL)-24 in mediating its antitumor activities. J. Mol. Signal. 8, 15 (2013). 24377906 10.1186/1750-2187-8-15 PMC3879428

[b22] Boukamp P. . Normal keratinization in a spontaneously immortalized aneuploid human keratinocyte cell line. J Cell Biol. **106**, 761–71 (1988). 2450098 10.1083/jcb.106.3.761 PMC2115116

[b23] Popp S. . Genetic characterization of a human skin carcinoma progression model: from primary tumor to metastasis. J. Invest. Dermatol. 115, 1095–103 (2000). 11121147 10.1046/j.1523-1747.2000.00173.x

[b24] Bertolotto C. . Microphthalmia gene product as a signal transducer in cAMP-induced differentiation of melanocytes. J. Cell Biol. 142, 827–35 (1998). 9700169 10.1083/jcb.142.3.827 PMC2148160

[b25] Campeau E. . 2009. A versatile viral system for expression and depletion of proteins in mammalian cells. PLoS One 4, e6529 (2009). 19657394 10.1371/journal.pone.0006529 PMC2717805

[b26] Kuhlbrodt K. . Functional analysis of Sox10 mutations found in human Waardenburg-Hirschsprung patients. J. Biol. Chem. 273, 23033–8 (1998). 9722528 10.1074/jbc.273.36.23033

[b27] Li X., Glubrecht D. D. & Godbout R. AP2 transcription factor induces apoptosis in retinoblastoma cells. Genes Chromosomes Cancer 49, 819–30 (2010). 20607706 10.1002/gcc.20790 PMC3726383

[b28] Bustin S. A. . The MIQE guidelines: minimum information for publication of quantitative real-time PCR experiments. 2009. Clin. Chem. 55, 611–22 (2009). 19246619 10.1373/clinchem.2008.112797

