# Supplementary material for: Loss of tumorigenic potential upon transdifferentiation from keratinocytic into melanocytic lineage
Source: Sci Rep. 2016 Jul 8;6:28891. doi: 10.1038/srep28891 (PMC4937495; doi:10.1038/srep28891)
Supplement: Supplementary Information [file srep28891-s1.pdf]

Supplement

Loss of tumorigenic potential upon transdifferentiation from keratinocytic into melanocytic lineage

Sabrina Fehrenbach 1,2, Daniel Novak 1,2, Mathias Bernhardt 1,2, Lionel Larribere 1,2, Petra Boukamp 3,4, Viktor Umansky 1,2, Jochen Utikal 1,2,*

1 Skin Cancer Unit, German Cancer Research Center (DKFZ), 69121 Heidelberg, Germany

2 Department of Dermatology, Venereology and Allergology, University Medical Center Mannheim, Ruprecht-Karl University of Heidelberg, 68167 Mannheim, Germany

3 Genetics of Skin Carcinogenesis, German Cancer Research Center (DKFZ), 69121 Heidelberg, Germany

4 IUF–Leibniz Research Institute for Environmental Medicine, 40021 Düsseldorf, Germany.

Supplementary Figure 1: A) Scheme of lentiviral vector constructs. Vectors carrying the transdifferentiation factors also harbor RFP to visualize transgene expression. The MITF promoter was linked to GFP to make endogenous MITF expression visible with this reporter construct. B) Validation of the functionality of the melanocyte-specific reporter construct. 12 days after infection with the reporter construct reporter activity was observed in melanocytes as indicated by strong GFP signal. No activity of the melanocyte-specific reporter construct was detectable in HaCaT cells and MET-4 cells 12 days after infection. C) Endogenous (endo) and total expression of the four transcription factors MITF, SOX10, SOX9 and LEF1 with and without induction with doxycycline in MET-4 cells. Error bars depict the SD of three independent experiments. P values were calculated by two-tailed, paired sample t-test. Asterisks indicate t-test p-value <0.05 (** p-value <0.01, *** p-value <0.005).

Supplementary Figure 2: A) MITF alone is not sufficient to induce neither MITF reporter activity nor morphological changes in MET-4 cells. MET-4 cells could not be cultured in the melanocyte medium MCDB. These cells died within a week in the MCDB medium. B) Strong RPF signal was observed in MET-4 cells after transfection with the transdifferentiation factors MITF, LEF-1, SOX9 and SOX10 and induction with doxycycline. After doxycycline withdrawal RFP signal was absent indicating a complete shutdown of transgene expression. However, the cells retained their changed morphology.

Supplementary Figure 3: Overview of senescence marker expression in MET-4 and MT-MET-4 (TD) cells

Supplementary Figure 4: IL-24 mRNA expression in MET-4 cells and MT-MET-4 cells cultured with and without TPA.

Supplementary Figure 1

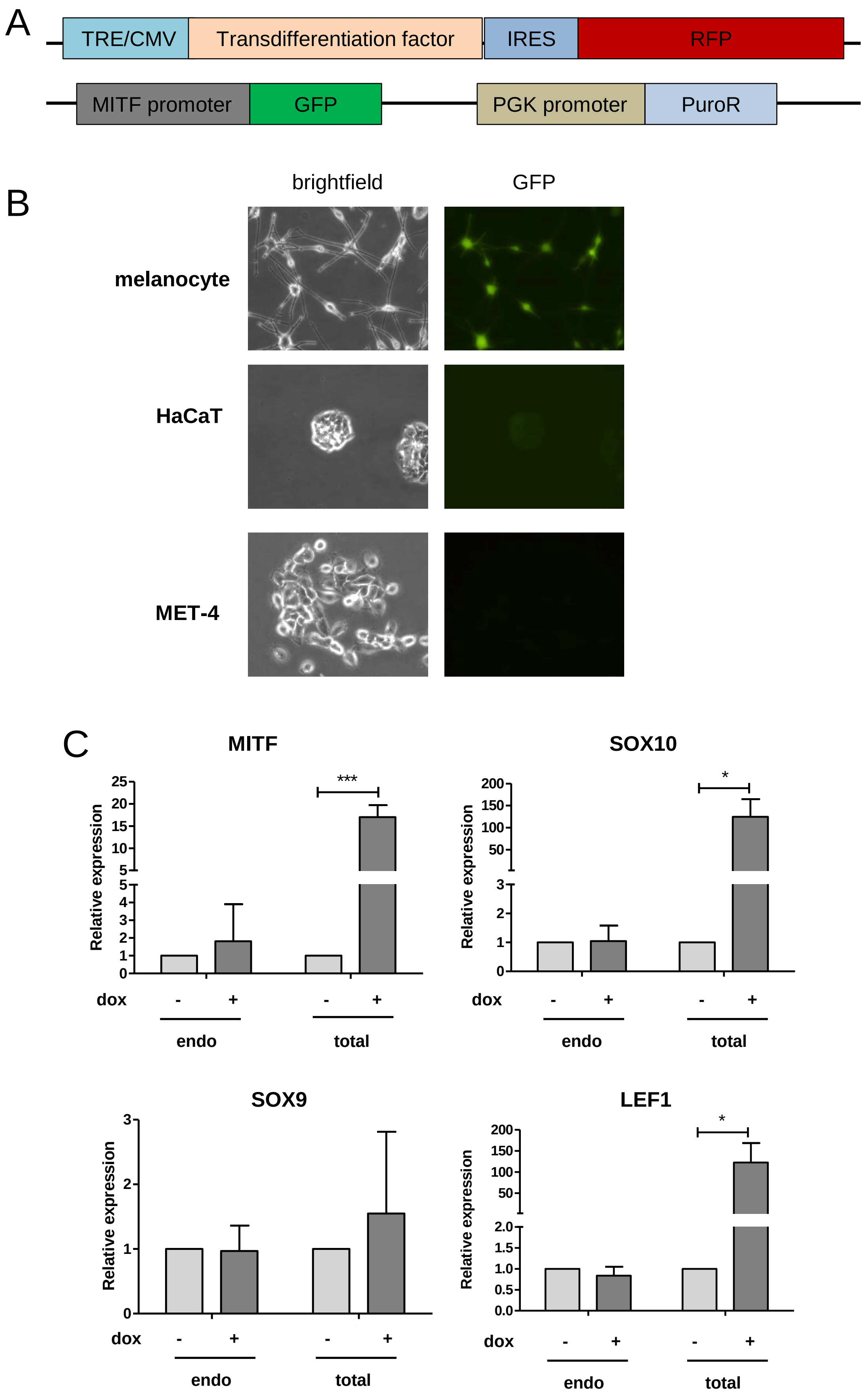

Supplementary Figure 2

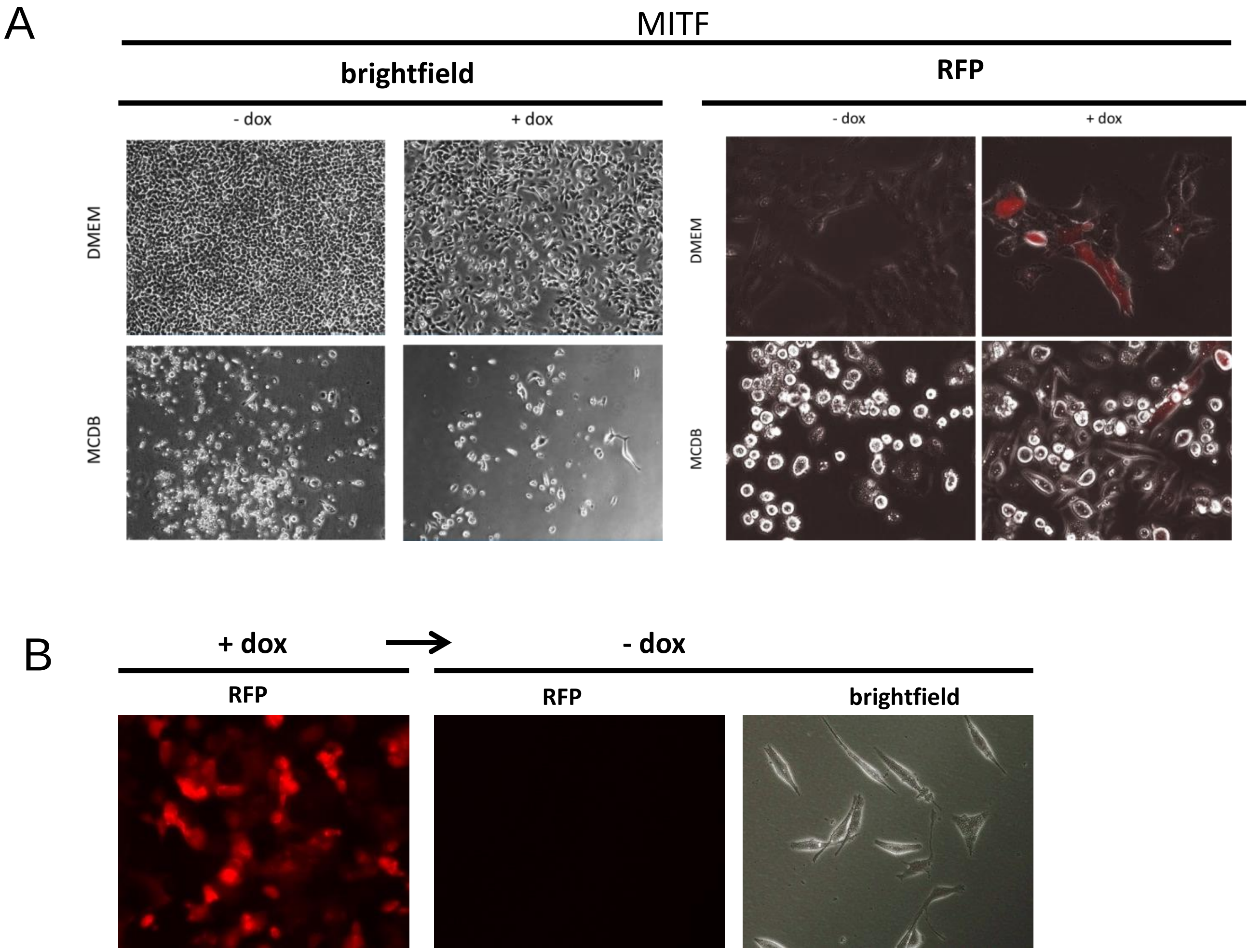

Supplementary Figure 3

A

	-1<FC<+1	MET4	TD
cellular senescence-associated genes (GO:0090398)	ABL1	9,31	9,46
	ARNTL	6,75	6,96
	BCL2L12	7,16	7,15
	BCL6	6,54	6,54
	BMPR1A	6,71	6,54
	C2orf40	6,47	6,48
	CALR	11,24	11,09
	CAV1	13,00	12,94
	CDK6	10,91	10,63
	CDKN1A	6,53	6,58
	CDKN2A	6,53	6,69
	H2AFX	9,92	10,19
	HMGA1	6,74	6,87
	HMGA2	6,76	7,15
	HRAS	9,79	9,18
	ING2	9,08	9,35
	KAT6A	8,15	8,29
	MAGEA2	6,44	6,41
	MAP2K1	10,44	9,63
	MAPK14	6,89	7,44
	MAPKAPK5	9,07	9,82
	NEK4	6,69	6,82
	NEK6	9,15	8,63
	NSMCE2	9,49	9,09
	NUAK1	10,24	9,78
	OPA1	8,28	8,74
	PLA2R1	6,91	6,51
	PML	7,03	6,77
	PNPT1	7,37	7,90
	PRKCD	9,20	8,40
	PRKDC	9,61	10,64
	PRMT6	8,46	8,96
	RSL1D1	10,34	11,12
	SIRT1	7,87	7,47
	SMC5	6,57	6,48
	SMC6	7,03	7,27
	SRF	10,37	10,16
	TBX2	6,45	6,69
	TBX3	6,59	6,66
	TERF2	7,91	8,08
	TP53	7,79	7,96
	TWIST1	9,40	10,32
	ULK3	6,82	6,96
	VASH1	6,82	6,51
	WNT16	6,68	6,60
	ZKSCAN3	7,24	7,41
	ZNF277	7,62	7,77

B

	-1<FC<+1	MET4	TD
SASP-associated genes	CCL13	6,61	6,84
	CCL2	6,49	6,54
	CCL27	6,63	6,59
	CCL3	6,62	6,60
	CCL8	6,67	6,61
	CSF3	6,61	6,96
	CXCL12	6,46	6,62
	CXCL13	6,52	6,53
	CXCL2	9,31	10,23
	IFNG	6,41	6,44
	IGFBP7	12,06	11,78
	IL13	6,42	6,45
	IL1B	10,31	10,57
	IL7	6,55	6,52
	IL8	8,37	8,51

Supplementary Figure 4

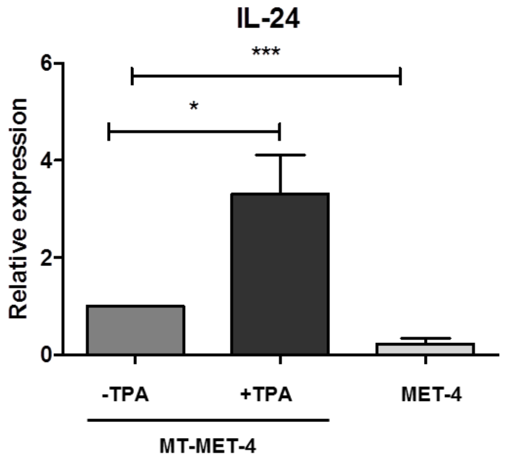

Suppl. Table 1: Candidate transcription factors used for transdifferentiation from keratinocytic into melanocytic lineage

**Candidate transcription factors for transdifferentiation**			
MITF-M	NM_000248.3	cMET	XM_011516223.1
SOX2	NM_003106.3	TFAP2A	NM_003220.2
SOX5	XM_011520846.1	NFIX	NM_002501.3
SOX9	NM_000346.3	IRF4	NM_002460.3
SOX10	NM_006941.3	ETS1	NM_005238.3
LEF1	NM_016269.4	FOSB	NM_006732.2
β-catenin	XM_005264886.2	HAND1	NM_004821.2
PAX3A	NM_000438.5	HES1	NM_005524.3
PAX3B	NM_013942.4	HOXB7	NM_004502.3
PAX3C	NM_181457.3	IFI16	NM_005531.2
PAX3D	NM_181458.3	KLF9	NM_001206.2
PAX3I	NM_001127366.2	PITX1	NM_002653.4
SNAI2	NM_003068.4		

Suppl. Table 2: Q-PCR primers

Q-PCR Primer	Forward primer	Reverse primer
**MITF**	GCTCACAGCGTGTATTTTTCC	GCTCACAGCGTGTATTTTTCC
**DCT**	GGTTCCTTTCTTCCCTCCAG	GGTTCCTTTCTTCCCTCCAG
**TRP1**	AGCAGTAGTTGGCGCTTTGT	AGCAGTAGTTGGCGCTTTGT
**TYR**	TTGTACTGCCTGCTGTGGAG	TTGTACTGCCTGCTGTGGAG
**K10**	GCTGACCTGGAGATGCAAAT	GCTGACCTGGAGATGCAAAT
**K14**	AGGAGGTCACATCTCTGGATGACTG	AGGAGGTCACATCTCTGGATGACTG
**Integrin beta 4**	CTGTACCCGTATTGCGACT	CTGTACCCGTATTGCGACT
**Integrin alpha 6**	GCTGGTTATAATCCTTCAATATCAATTGT	GCTGGTTATAATCCTTCAATATCAATTGT
**Loricrin**	GGAGTTGGAGGTGTTTTCCA	GGAGTTGGAGGTGTTTTCCA
**Involucrin**	CTCCATGTGTCATGGGATATG	CTCCATGTGTCATGGGATATG
**IL-24**	GACTTTAGCCAGCAGACCCTT	GGTTGCAGTTGTGACACGAT
**18S**	GAGGATGAGGTGGAACGTGT	GAGGATGAGGTGGAACGTGT
**MITF endo**	ACCGTCTCTCACTGGATTGG	CGTTGGGCTTGCTGTATGTG
**MITF total**	TGCCTGTCTCGGGAAACTTG	CCAGTGCTCTTGCTTCAGAC
**SOX9 endo**	AGACCTTTGGGCTGCCTTAT	TAGCCTCCCTCACTCCAAGA
**SOX9 total**	CTGAGCAGCGACGTCATCTC	GTTGGGCGGCAGGTACTG
**SOX10 endo**	GGCTTTCTGTCTGGCTCACT	TAGAGGGTCATTCCTGGGGG
**SOX10 total**	AGCCCAGGTGAAGACAGAGA	ATAGGGTCCTGAGGGCTGAT
**LEF1 endo**	GCATCAGGTACAGGTCCAAGA	TCTCCAGAAGAGGTCCTGGG
**LEF1 total**	AGAGCGAATGTCGTTGCTGA	TCTTGGACCTGTACCTGATGC
